# Role of the SDF-1/CXCR4 signaling pathway in cartilage and subchondral bone in temporomandibular joint osteoarthritis induced by overloaded functional orthopedics in rats

**DOI:** 10.1186/s13018-020-01860-x

**Published:** 2020-08-14

**Authors:** Jing Yang, Yazhen Li, Ying Liu, Qiang Zhang, Qi Zhang, Junbo Chen, Xiao Yan, Xiao Yuan

**Affiliations:** 1 Department of Orthodontics, Affiliated Hospital of Qingdao University, Qingdao University, Jiangsu Road No. 16, Qingdao, 266000 Shandong People’s Republic of China; 2 Qingdao Stomatological Hospital, Qingdao, Shandong People’s Republic of China; 3 West China School of Stomatology, Sichuan University, Chengdu, Sichuan People’s Republic of China; 4 Second Affiliated Hospital of Shandong University, Shandong University, Jinan, Shandong People’s Republic of China; 5 School of Stomatology, Qingdao University, Qingdao, Shandong People’s Republic of China

**Keywords:** Temporomandibular joint osteoarthritis; Overloaded functional orthopedics; Subchondral bone; SDF-1/CXCR4

## Abstract

**Objectives:**

To (i) use a mandibular advancement appliance in rats to investigate the role of the stromal cell-derived factor/CXC receptor 4 (SDF-1/CXCR4) signaling pathway in temporomandibular joint osteoarthritis (TMJ OA) induced by overloaded functional orthopedics (OFO) and (ii) provide a cellular and molecular basis for efficacious treatment of skeletal class-II malocclusion and avoidance of TMJ OA.

**Method:**

Male Sprague-Dawley rats (6 weeks) were divided randomly into control + normal saline (NS), EXP + ADM3100 (SDF-1 antagonist), EXP + NS, and control + ADM3100 groups. Changes in articular cartilage and subchondral bone after TMJ OA in these four groups were observed by hematoxylin and eosin (H&E), immunofluorescence double staining (IDS), Safranin-O staining, immunohistochemical (IHC) staining, real-time polymerase chain reaction, and micro-computed tomography at 2, 4, and 8 weeks.

**Results:**

OFO led to increased expression of SDF-1, CXCR4, and matrix metalloproteinase (MMP) 13 and decreased expression of collagen II. The thickness of the hypertrophic cartilage layer was reduced at 4 weeks in the EXP + NS group, and damage to subchondral bone was observed at 2 weeks. Using ADM3100 to inhibit SDF-1 signaling could attenuate expression of MMP13, cartilage damage, and osteoblast differentiation. IDS showed that the areas of expression of SDF-1 and OSX in subchondral bone overlapped.

**Conclusions:**

Overloaded functional orthopedics (OFO) induced TMJ OA. The destruction of subchondral bone in TMJ OA caused by OFO occurred before damage to cartilage. SDF-1/CXCR4 may induce the osteogenic differentiation and cause cartilage degradation in TMJ OA caused by OFO.

## Introduction

Class-II malocclusion is characterized by a convex facial profile, protrusive and everted lips, and a deep mentolabial fold. Mandibular retrusion is considered to be a main factor that contributes to a class-II malocclusion [1]. Consequently, functional therapy is an effective strategy to coordinate the relationship between maxillary and mandibular structures in growing patients, which is based on adaptive remodeling of the temporomandibular joint (TMJ) [2].

Growth of the mandibular condyle involves endochondral ossification; mature chondroblasts and the extracellular matrix are replaced by newly formed bone [3]. Condylar cartilage is present throughout post-natal life and retains the capacity for growth and remodeling in response to mechanical stimuli or changes in mandibular position [4]. Biomechanical stimuli produced by mandibular advancement solicit cellular and molecular changes in mandibular condyles, which alter the phenotypic as well as morphologic changes of chondrocytes, and subsequently, the transition from chondrogenesis to osteogenesis is accomplished [5]. During treatment based on functional orthopedics, the TMJ maintains a balance between anabolism and catabolism with regard to adaptive remodeling [6]. Once biomechanical stimuli exceed the capacity for adaptive remolding of the TMJ, production of inflammatory factors and matrix metalloproteinases (MPPs) will increase, and symptoms of a temporomandibular disorder will be detected: this is called “temporomandibular joint osteoarthritis” (TMJ OA) induced by overloaded functional orthopedics (OFO) [7]. Exploring the mechanism of TMJ OA induced by OFO could provide new approaches for clinical treatment of class-II malocclusions.

TMJ OA is characterized by a slow, progressive degradation of articular cartilage and changes in subchondral bone [8]. The structural and functional integrity of condylar cartilage is dependent upon interactions between subchondral bone and the surrounding soft tissues [9]. Studies have focused mostly on aberrant remodeling responses in condylar cartilage by occlusal abnormalities in different animals, and models of mandibular advancement have been reported [10]. In recent years, scholars have stated that dense, stiff bone can alter the biomechanics and mechanical properties of joints. Accumulating evidence suggests that changes in subchondral bone have critical roles in TMJ OA [11]. Funck-Brentano and colleagues reported that abnormalities in subchondral bone can induce OA in mice with initially normal cartilage [12]. Although an increasing number of scholars have focused on the mechanism of TMJ OA, the relationship between articular cartilage and subchondral bone in TMJ OA induced by OFO merits further exploration.

Chemokines are small soluble peptides that regulate the movement, morphology, proliferation, differentiation, and other activities of cells [13]. Stromal cell-derived factor (SDF)-1 belongs to the CXC subfamily of chemokines, which are involved in the activation, differentiation, and migration of immune cells due to binding to CXC receptor 4 (CXCR4) [14]. Hosogane and coworkers reported that SDF-1 may contribute to cartilage destruction during arthritis by activating the extracellular signal-regulated kinase signaling pathway and downstream transcription factors [15]. Chen and colleagues reported that SDF-1/CXCR4 can promote the development and differentiation of osteoblasts and induce abnormal changes of subchondral bone in OA [16]. Furthermore, an animal study by Wei and coworkers showed that cartilage degeneration can be alleviated by using AMD3100 (a CXCR4 antagonist of the SDF-1 receptor) [17]. Consequently, we hypothesized that obvious abnormalities in the condylar subchondral bone exhibited by SDF-1 could play an important part in TMJ OA induced by OFO.

In the present study, we used a mandibular advancement appliance in rats to test the changes in subchondral bone and cartilage degradation by morphologic observation of the mandibular condyle. We also measured mRNA and protein levels of SDF-1, CXCR4, MMP13, and collagen II. An immunofluorescence double staining (IDS) method for osterix (OSX) and SDF-1 was employed to illustrate the role of SDF-1/CXCR4 in TMJ OA induced by OFO.

## Materials and methods

### Ethical approval of the study protocol

Animal care and all procedures involving animals were in accordance with the guidelines of the Animal Research Committee of Qingdao University (Qingdao, China). The study protocol was approved by Qingdao University.

### Experimental animals and grouping

One-hundred and eight Sprague-Dawley (SD) rats (6 weeks; 60–180 g) were provided by the Animal Center of Qingdao University.

Rats were allocated randomly into four groups (*n* = 27): control + normal saline (NS), experimental (EXP) + NS, EXP + inhibitor (AMD3100), and EXP + AMD3100 groups; each group was subdivided into three time points (*n* = 9).

### Experimental procedures

**Fig. 1:**
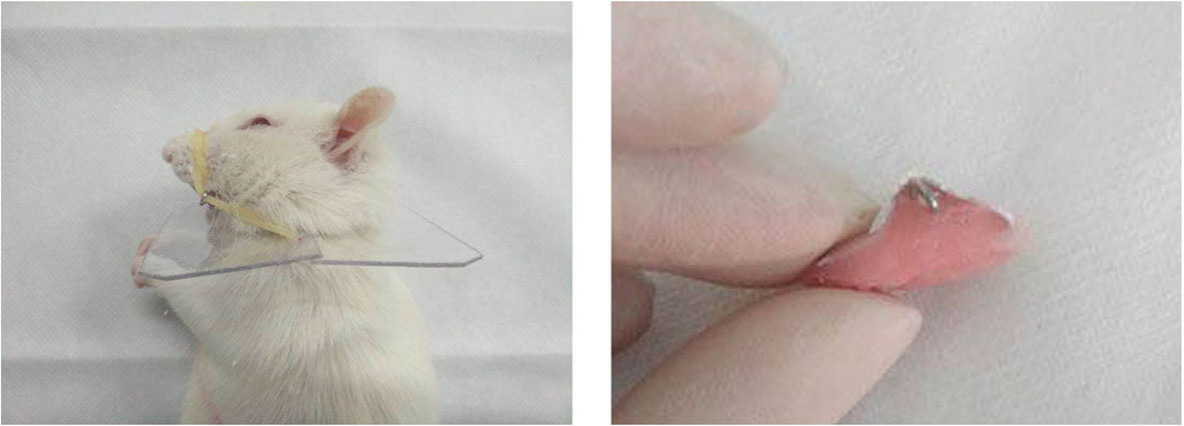
The removable functional appliance used to create forward mandibular advancement. It is composed of three main parts: an inclined guide plate, upper-incision crown, and extraoral auxiliary retention device. This leads to 4 mm of incisal open bite in the vertical dimension and 3 mm mandibular advancement in the sagittal dimension

Anesthesia was induced by 10% chloral hydrate (0.3 mL/kg bodyweight, i.p.). The removable functional appliance designed by Petrovic and colleagues [18] was fitted in rats in the EXP group to create forward mandibular advancement (Fig. 1). This appliance is composed of three main parts: an inclined guide plate, an upper-incision crown, and an extraoral auxiliary retention device. The inclined guide plate is made of methyl methacrylate and an aluminum alloy sheet (thickness, 0.5 mm). The labial thickness of the upper-incision crown is ~ 1 mm. The angle between the inclined guide plate and occlusal plane is designed to be 30–40°. The retentive part comprises the upper-incision crown and an extraoral auxiliary retention device. There are retention arms on both sides of the appliance. To prevent the removable appliance from loosening, the appliance was fixed to the nasal maxillary complex through a rubber band. A Plexiglass™ baffle with a border length of ~ 5 cm was obtained; the center of the baffle had a hole of diameter ~ 1.8 cm. The baffle was placed on the neck of rats to assist retention. When rats closed their jaw, 4 mm of incisal open bite in the vertical dimension and 3 mm mandibular advancement in the sagittal dimension were rebuilt. Appliances were checked every other day. Control groups underwent the same procedure but without the removable functional appliance. AMD3100 is an antagonist of SDF-1. Rats in the EXP + AMD3100 and control + AMD3100 groups were injected bilaterally with 40 μL (1μg/μL) of AMD3100 (diluted in NS) into the TMJ area, and rats in the EXP + NS and control + NS groups were injected bilaterally with 40 μL of NS into the TMJ area once weekly for 8 weeks.

After the experimental procedure, animals with the appliance were fed a soft diet for 3 days to allow them to get used to the appliance. Rats in all groups received a standard diet from 4 days after the experimental procedure.

### Tissue preparation

Animals were sacrificed 2, 4, or 8 weeks after the beginning of the experimental procedure. Similar to a study by Wang, differences in degradation from the left and right sides were not observed [19]. Blocks of TMJ tissue from the left side of six mice of each group were fixed with 4.0% paraformaldehyde at 4 °C for 24 h and decalcified with 10% ethylenediaminetetraacetic acid solution for 4 weeks at room temperature. TMJs were dehydrated in a series of ethanol solutions and embedded in paraffin wax. Serial sections of thickness 5 mm were cut through the TMJ in the sagittal plane using a rotary microtome. These sections were selected randomly for hematoxylin and eosin (H&E) staining; Safranin-O staining; immunohistochemical (IHC) staining of SDF-1, collagen II, MMP13, and CXCR4; and IDS of SDF-1 and OSX. The other three left-side blocks of TMJ tissue were separated from the mandible and were fixed immediately with 4% glutaraldehyde for 24 h at 4 °C; these blocks were used for micro-computed tomography (micro-CT). All condylar heads of the right-side joints in each group were isolated and preserved at − 80 °C for real-time polymerase chain reaction (PCR) analyses.

### Histochemical and IHC staining

**Fig. 2:**
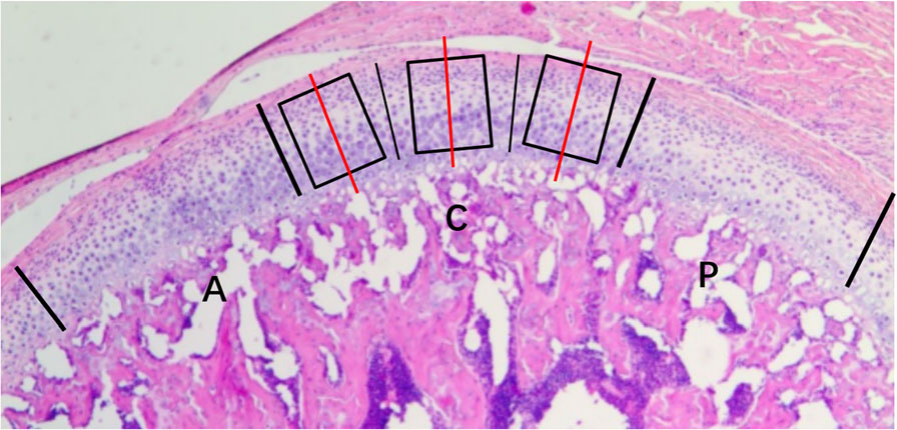
The mandibular condylar cartilage was divided sagittally into three equal parts according to the articular surface: A, anterior part; C, most central part; and P, posterior part. Black line represents the midpoint of anterior, central, and posterior parts. Three squares covering all hypertrophic layers were applied at the quarter points of the center thirds of condylar cartilage. Red line represents median line. The cartilage thickness in the central third was determined as the average length of the three red lines in each third

H&E staining and Safranin-O staining were undertaken to determine histochemical and proteoglycan changes in condyles. IHC staining involved use of four primary antibodies obtained from Abcam (Cambridge, UK): anti-SDF-1 (rabbit polyclonal; 1:50 dilution), anti-CXCR4 (rabbit polyclonal; 1:50), anti-MMP13 (rabbit polyclonal; 1:50), and anti-collagen II (1:50). The avidin-biotin complex (ABC) IHC staining protocol reported by Wang and colleagues was employed [20]. Negative controls were stained with non-immune serum instead of the primary antibody. Images of H&E, Safranin-O, and IHC staining were acquired using a DFC490 system (Leica, Wetzlar, Germany), as described previously [21]. Briefly, the surface of condylar cartilage was divided equally into anterior, central, and posterior thirds between the anterior and posterior attachment points of the joint disk to the condyle. Three squares (300 mm × 300 mm) covering all hypertrophic layers were applied at the quarter points of the center thirds of condylar cartilage. The cartilage thickness in the central third was determined as the average length of the three short lines in each third (Fig. 2). The areas of positive cells were measured with a computer-assisted image-analyses system (Qwin Plus; Leica). The average value of six squares was calculated for statistical analyses. OA severity was assessed using the Osteoarthritis Research Society International (OARSI) [22] score under light microscopy by staff from the Histology or Pathology Departments of Qingdao University.

### IDS

Expression of SDF-1 and OSX on the cartilage and subchondral bone of condyles was measured using IDS in the EXP, EXP + inhibitor, EXP + NS, control + inhibitor, and control groups 2, 4, or 8 weeks after the beginning of the experimental procedure. IDS was carried out with a three-step ABC method, as described previously [3]. For IDS, sections were incubated with a primary antibody against OSX monoclonal antibody (anti-rabbit; 1:100) diluted in phosphate-buffered saline at 4 °C overnight. After washing, sections were incubated with the corresponding secondary antibody (goat anti-rabbit; 1:300; Google Biological Technology, Wuhan, China). Next, sections were incubated with an anti-SDF-1 primary monoclonal antibody (anti-mouse; 1:100; Abcam) and a secondary antibody (488 goat anti-mouse; 1:400; Google Biological Technology). Sections were imaged under a confocal laser scanning microscope (LSM510META; Zeiss, Jena, Germany). Positive integrated optical densities (IODs) of SDF-1 or OSX were evaluated using Image-Pro Plus 6.0 (Media Cybernetics, Rockville, MD, USA) whereby IOD = positive area in immunofluorescence image × optical density. A higher IOD corresponded to higher expression of SDF-1 or OXS.

### RNA extraction and real-time PCR

Samples of condylar cartilage from each group were homogenized. Total RNA was extracted using TriPure™ Isolation Reagent (Roche, Basel, Switzerland). The primers for the target genes are listed in Table 1. Gene expression was measured with a real-time PCR machine (7500; Applied Biosystems, Foster City, CA, USA) with glyceraldehyde-3-phosphate dehydrogenase (GAPDH) as the internal control. Each experiment was carried out three times, and the mean values were calculated. Expression of target genes, relative to that of GAPDH, was calculated using the 2−^△△Ct^ method. Results were calculated as the relative quantification of the target gene compared with that in the control group.

**Table 1:** Sequences of primers of target genes used for real-time PCR

Target gene	Upstream primer sequence	Downstream primer sequence
Cxcl12	ATATTCATCCGTGCCCTCG	GCAATGCCACCACCTGTAAC
Cxcr4	GTCTATGTGGGTGTCTGGAT	ATGATGTGCTGGAACTGG
Col2a1	AAGAAGCACATCTGGTTTGGA	CAGTGGACAGTAGACGGAGGA
Mmp13	TGATAGACTCCGAGAAATGC	GTTTGGGACCATTTGAGTG
Gapdh	TTCAACGGCACAGTCAAGG	CTCAGCACCAGCATCACC

### Micro-CT

Specimens were imaged and analyzed using a micro-CT system (Latheta LCT 200, Hitachi, Tokyo, Japan). Two cubic regions of interest (ROIs; 0.25 × 0.25 × 0.25 mm) were selected at the midpoint of the central and posterior condyle of subchondral bone. These ROIs were used to measure and compare microstructural parameters (the ratio of bone volume to tissue volume (BV:TV), trabecular thickness (Tb.Th), trabecular separation (Tb.Sp), trabecular number (Tb.N), ratio of bone surface to bone volume (BS:BV), bone mineral density (BMD)) between different experimental groups at three time points.

## Results

### Overloaded functional orthopedics induced TMJ OA

**Fig. 3:**
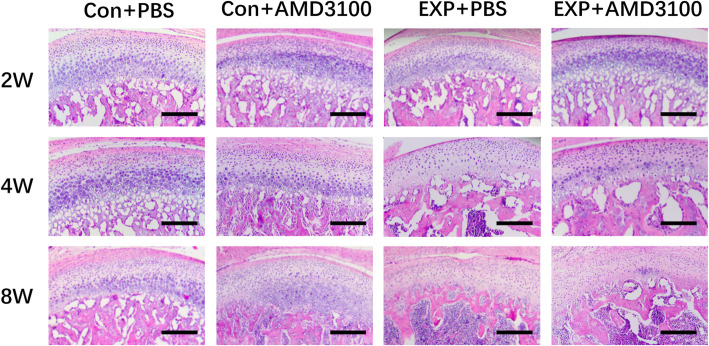
Histology of the mandibular condyle in the control + NS, EXP + NS, EXP + AMD3100, and control + AMD3100 groups. Scale = 100 μm

**Fig. 4:**
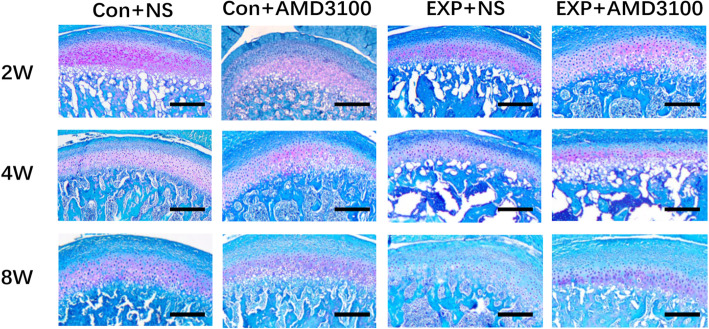
Safranin-O staining of the mandibular condyle in the control + NS, EXP + NS, EXP + AMD3100, and control + AMD3100 groups. Scale = 100 μm

**Fig. 5:**
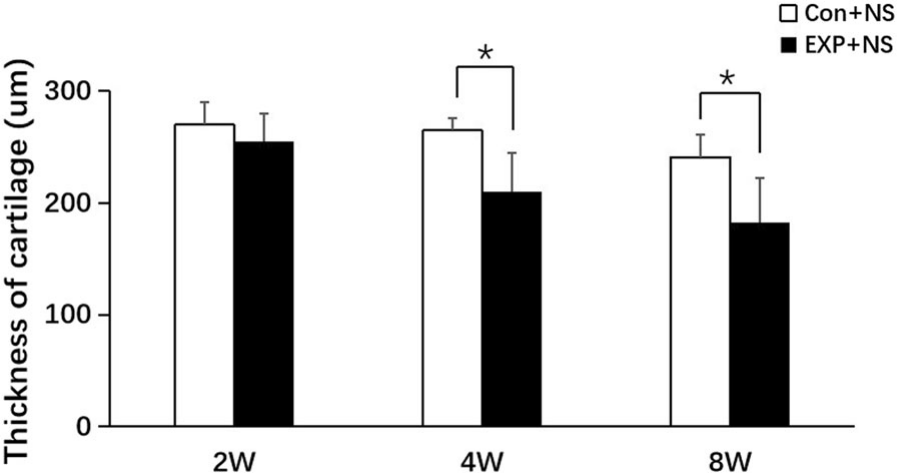
Thickness of condylar cartilage in the control + NS group and EXP + NS group at 2, 4, and 8 weeks. All groups were compared with the control group,*P < 0.05

**Fig. 6:**
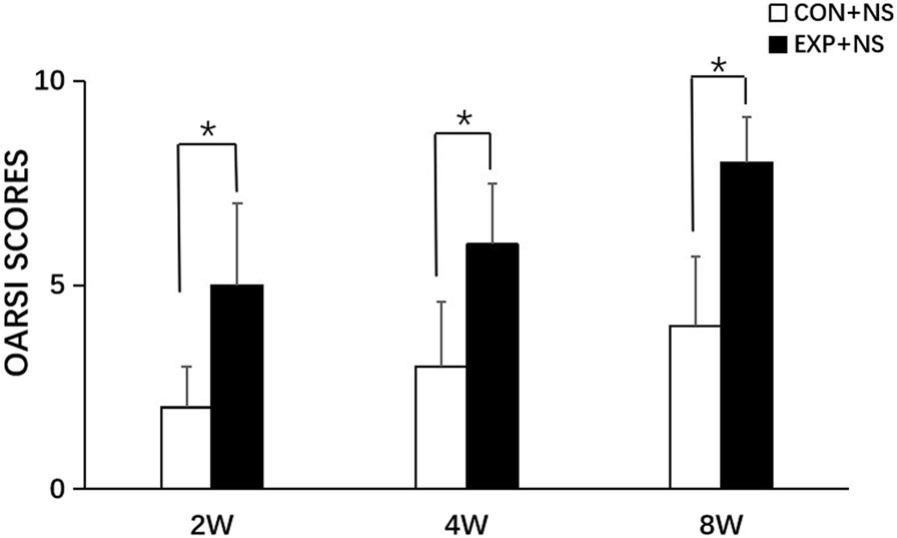
The OARSI score in the control + NS group and EXP + NS group at 2, 4, and 8 weeks. All groups were compared with the control group,*P < 0.05

In the control + NS group, the condylar cartilage appeared normal, fibrous, proliferative, and hypertrophic, and endochondral ossification layers had an abundant cartilage matrix. The group with forward mandibular advancement showed OA-like lesions in the middle and posterior thirds of condylar cartilage. Irregular cellular arrangements and cell-free areas were detected. Homogeneous eosinophilic lesions and local loss of proteoglycans were detected at 2 weeks, and worsened at 8 weeks, in the EXP + NS group (Fig. 3). The EXP + NS group showed less intense Safranin-O staining at 4 weeks, illustrating reduced levels of proteoglycans (Fig. 4). Degradation of articular cartilage was alleviated in the EXP + AMD3100 group, compared with the EXP + NS group. The thickness of the hypertrophic cartilage layer compared with the whole cartilage layer in the EXP + NS group was decreased 4 weeks compared with that in the control + NS group (*P* < 0.05) (Fig. 5). The OARSI score in the EXP + NS group was significantly higher than that in the control + NS group (*P* < 0.05) (Fig. 6).

### Inhibition of SDF-1 signaling increases subchondral bone absorption induced by overloaded functional orthopedics

**Fig. 7:**
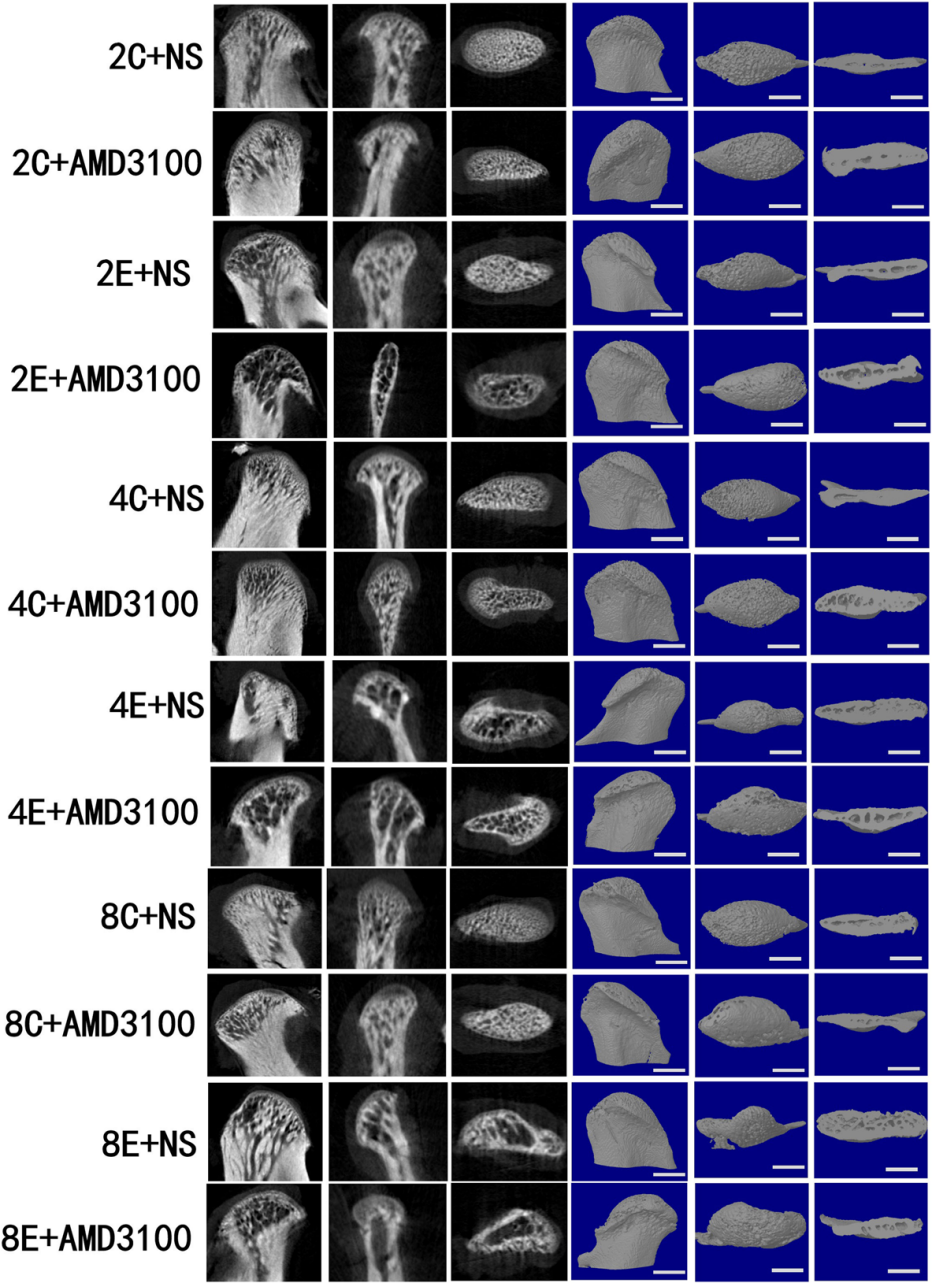
Microscopic slice of the TMJ condyle in rats. From the left to right are the median anomalous plane, coronal plane, horizontal plane, and three-dimensional map. Scale = 1 mm

**Fig. 8:**
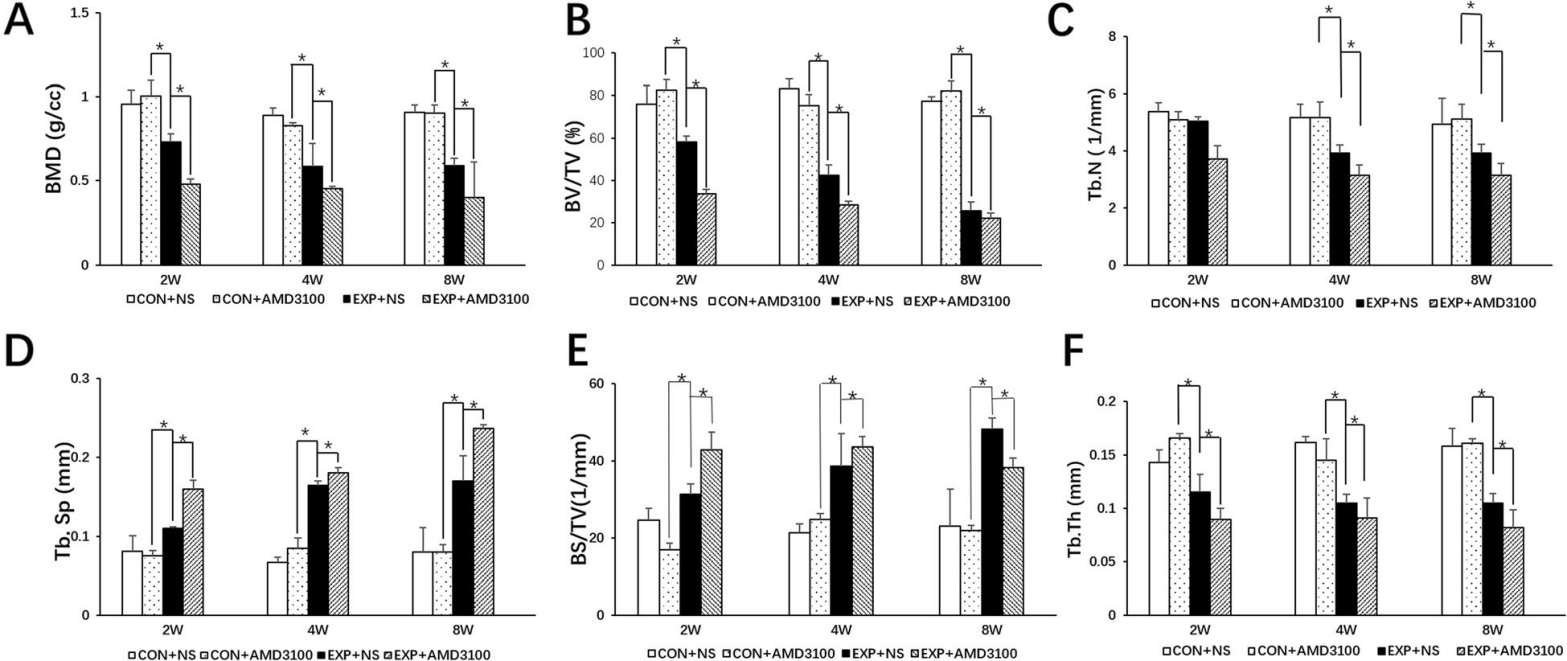
Micro-CT images to obtain the parameters of **a** bone mineral density (BMD), **b** ratio of bone volume to tissue volume (BV:TV), **c** number of bone trabeculae (Tb.N), **d** trabeculae spacing (Tb.Sp), **e** ratio of bone surface to bone volume (BS:BV), and **f** thickness of bone trabeculae (Tb.Th) in the condyles of subchondral bone in rats. All groups were compared with the control group,*P < 0.05

Three-dimensional (3D), 2D, and structural parameters of subchondral bone were tested by micro-CT (Fig. 7). BMD, BV:TV ratio, and Tb.Th in the EXP + NS group were lower than those in the control + NS group at 2, 4, and 8 weeks (*P* < 0.05 for all). Tb.N in the EXP + NS group was lower than that in the control + NS group at 4 and 8 weeks (*P* < 0.05 for all). Increases in Tb.Sp. and the BS:BV ratio were observed in the EXP + NS group at 2, 4, and 8 weeks compared with those in the control + NS group (*P* < 0.05 for all). There was no significant difference among the three time points tested in the control + AMD3100 group and control + NS group (*P* > 0.05). BMD, BV:TV ratio, Tb.N, and Tb.Th in the EXP + AMD3100 group were lower, but Tb.Sp and BS:BV ratio were higher than those of the EXP + NS group at 2, 4, and 8 weeks (*P* < 0.05 for all) (Fig. 8a–f).

### Inhibition of SDF-1 signaling upregulated the expression of Col II and downregulated the expression of SDF-1, CXCR4, and MMP13

**Fig. 9:**
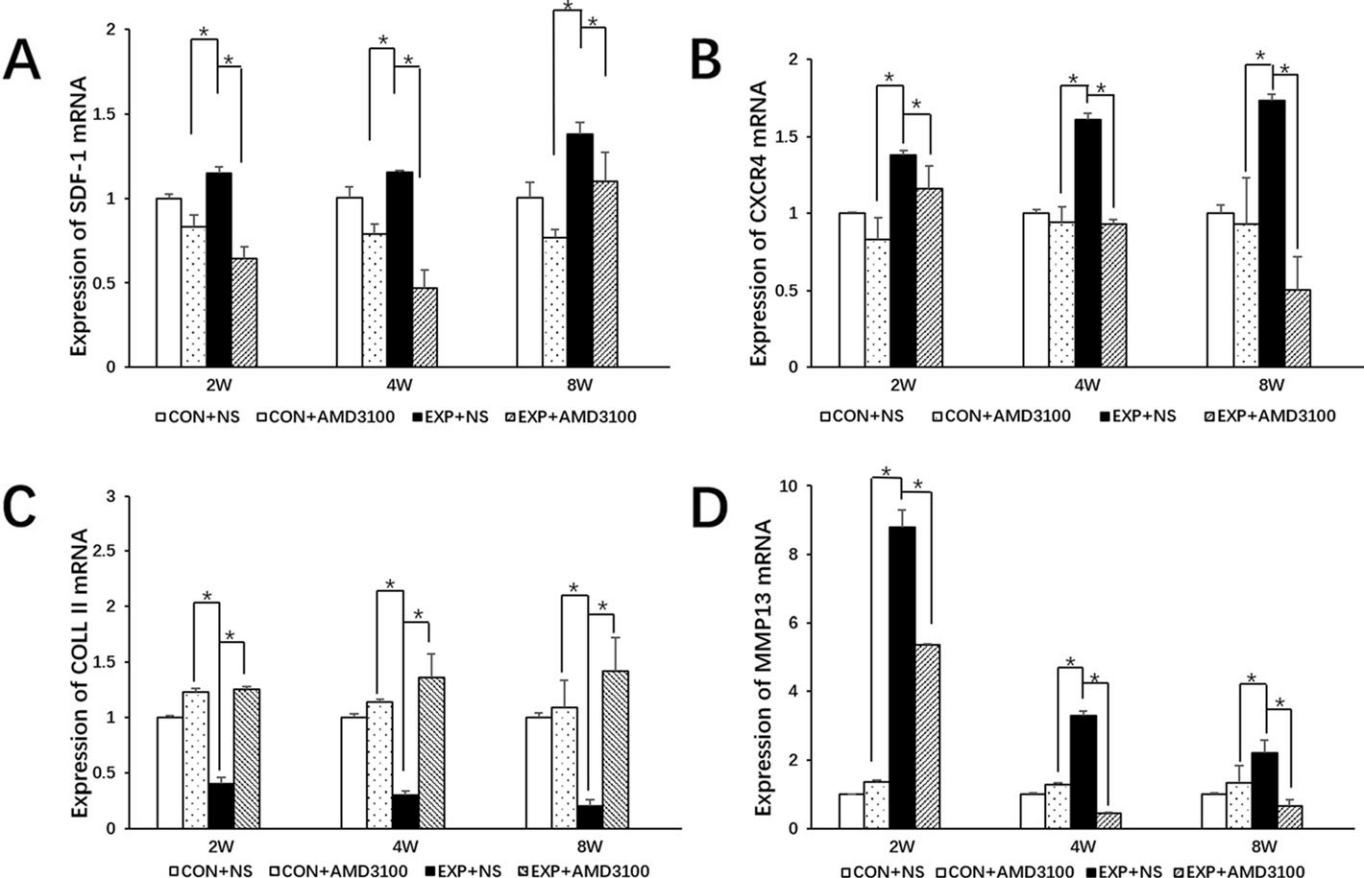
mRNA expression of **a** SDF-1, **b** CXCR4, **c** collagen II, and **d** MMP13 in the TMJ condylar cartilage of rats. All groups were compared with the control group,*P < 0.05

**Fig. 10:**
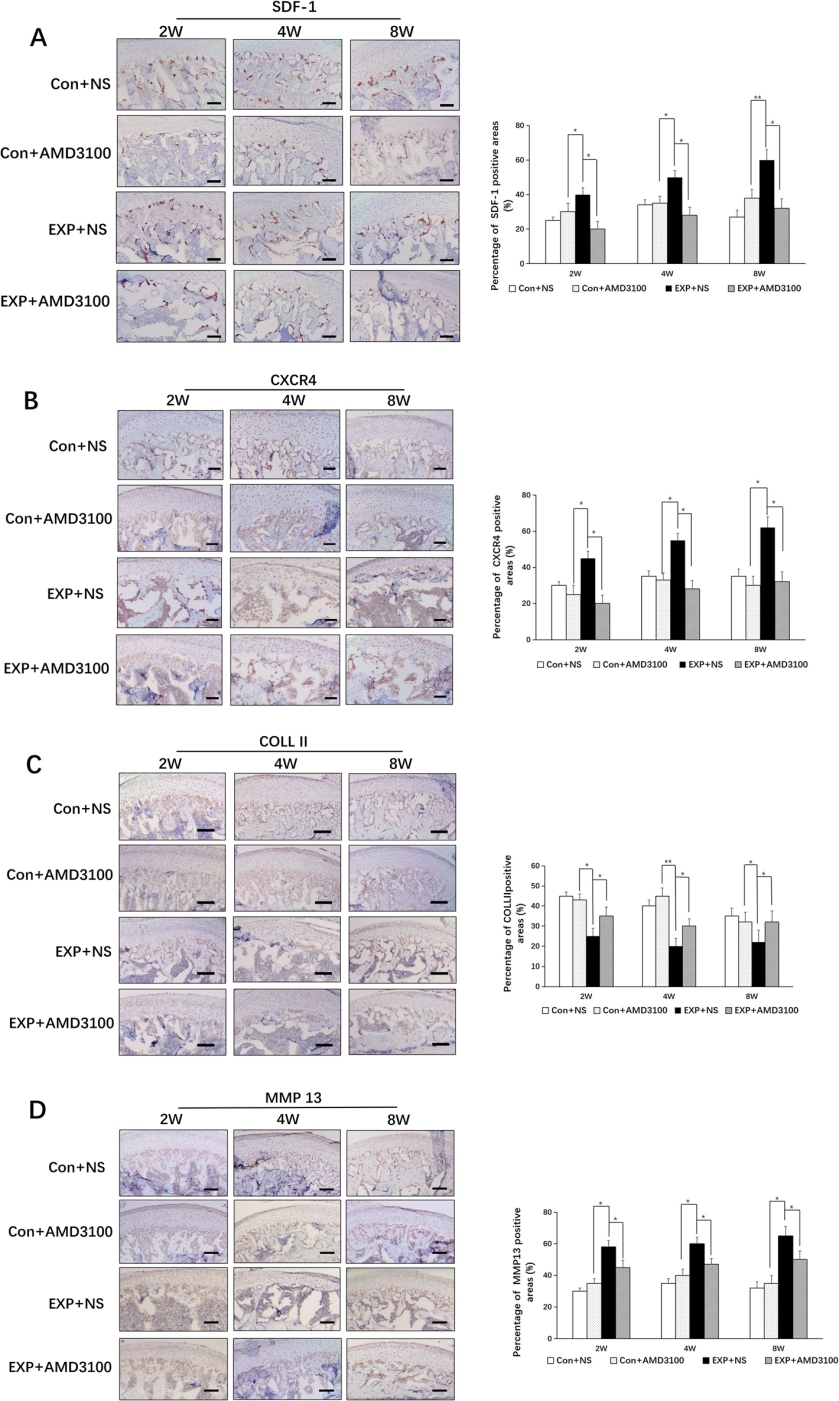
Immunohistochemical staining and expression of **a** SDF-1, **b** CXCR4, **c** collagen II, and **d** MMP13 in the condylar tissue of rats in the blank control group (control + NS), experimental group (EXP + NS), experimental injection group (model + AMD3100), and non-model injection group (control + AMD3100) at 2, 4, and 8 weeks. Scale = 100 μm. All groups were compared with the control group, *P < 0.05, **P < 0.01

mRNA expression of SDF-1, CXCR4, and MMP13 increased at 2, 4, and 8 weeks in the EXP + NS group compared with that in the control + NS group (*P* < 0.05 for both) (Fig. 9a–d). Expression of collagen II was decreased significantly in the EXP + NS group compared with that in the control + NS group at 2, 4, and 8 weeks (*P* < 0.05 for both). Expression of SDF-1 and CXCR4 was decreased at 2, 4, and 8 weeks in the EXP + AMD3100 group compared with that in the EXP + NS group (*P* < 0.05 for both). Increased expression of MMP13 was downregulated at 2, 4, and 8 weeks in the EXP + AMD3100 group compared with that in the EXP + NS group (*P* < 0.05 for both). Expression of collagen II was increased in the EXP + AMD3100 group compared with that in the EXP + NS group at 2, 4, and 8 weeks (*P* < 0.05 for both). In the control + AMD3100 group, expression of SDF-1, CXCR4, MMP13, and collagen II did not change significantly compared with that in the control + NS group. In the control + NS group, SDF-1 was seen predominantly in the bone-marrow area adjacent to the hypertrophic layer of cartilage (Fig. 10a). CXCR4, collagen II, and MMP13 showed high expression in the hypertrophic layer and low expression in proliferative layers (Fig. 10b, c). In the EXP + NS group, SDF-1 expression increased mainly in the bone marrow and hypertrophic layer at 2, 4, and 8 weeks compared with that in the control + NS group (*P* < 0.05 for all) (Fig. 10a). The percentage of CXCR4-positive chondrocytes increased in the proliferative and hypertrophic layers at 2, 4, and 8 weeks compared with that in the control + NS group (*P* < 0.05 for both) (Fig. 10b). Collagen II immunopositivity in the EXP + NS group was lower at 2, 4, and 8 weeks compared with that in the control + NS group (*P* < 0.05) (Fig. 10c). The percentage of MMP13-positive chondrocytes showed an increase in the EXP + NS group at 2, 4, and 8 weeks compared with that in the control + NS group (*P* < 0.05) (Fig. 10d). MMP13 expression was downregulated at 2, 4, and 8 weeks in the EXP + AMD3100 group compared with that in the EXP + NS group (*P* < 0.05 for both) (Fig. 10d). Expression of collagen II increased in the EXP + AMD3100 group compared with that in the EXP + NS group at 2, 4, and 8 weeks (*P* < 0.05) (Fig. 10c).

### SDF-1-positive cells in subchondral bone were derived mainly from osteoblasts

**Fig. 11:**
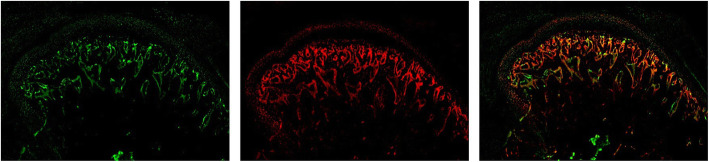
Immunofluorescence double staining (IDS) of OSX and SDF-1 in the experimental group (EXP + NS). Green fluorescence shows that SDF-1 is expressed mainly in the marrow of subchondral bone, and red represents the OSX distribution in subchondral bone. IDS shows that the areas of expression of SDF-1 and OSX in subchondral bone overlap

Green fluorescence of IDS represented SDF-1 expressed mainly in the marrow of subchondral bone, and red represented OSX distribution in subchondral bone. IDS showed that the areas of expression of SDF-1 and OSX in subchondral bone overlapped, suggesting that SDF-1-positive cells in subchondral bone were derived mainly from osteoblasts (Fig. 11).

## Discussion

Articular cartilage is bordered by the subchondral bone plate. Considerable evidence has demonstrated that channels and fissures between cartilage and bone could provide a route for biologic signals between these compartments [23]. Undulations of the tide mark, subchondral bone, and calcified cartilage can transform shear stresses into compressive and tensile stresses. The force transmitted to subchondral bone comprises compressive and tensile stresses, and ~ 30% of loads are attenuated by subchondral bone [24]. Consequently, subchondral bone is not only vital for cartilage metabolism; it may also be an important “shock absorber” [25]. Mechanical stress can induce remodeling of subchondral bone by initiating a cascade of physiologic responses by osteogenesis and osteoclasts [26]. The unbalanced activities between osteoclasts and osteoblasts that occur in remodeling of subchondral bone can induce disease [27]. Tensile stresses exerted on the condyle induced by functional orthopedics are a common form of treatment to solicit growth of condyles and the glenoid fossa to correct mandibular retraction in growing patients [28]. Several researchers have studied the mechanism of condylar adaptation to mandibular advancement [29]. However, the increasing prevalence of TMJ OA induced by the force applied during OFO focused our attention on the effect of OFO on articular cartilage and subchondral bone and to provide guidance for clinical practice.

We established an OFO model in rats using a mandibular advancement appliance. TMJ OA models were successfully induced by OFO. Spatial and temporal ultrastructural changes in subchondral bone were defined by micro-CT in the EXP + NS group with decreased BMD, BV:TV ratio, and Tb.Th and increased Tb.Sp and BS:BV ratio at 2 weeks. Although cartilage degradation (disorganized arrangement of cells and proteoglycan depletion) was observed at 2 weeks in the EXP + NS group, the thickness of the hypertrophic cartilage layer decreased at 4 weeks in the EXP + NS group. Some researchers have studied that damage to the subchondral bone plate and cartilage leads to direct contact between cartilage and bone, which elicits communication between them. They also found that more catabolic factors are crossing the chondro-osseous junction from subchondral bone to cartilage and promote cartilage damage [30]. Our study observed that only subchondral bone at the early stage of OA induced by OFO may proceed to microscopic cartilage damage at later stages. The possible reason may be that in our study, we used TMJ condyles of young rats, their cartilage and the subchondral bone marrow contact directly, and cartilage and subchondral bone do not need to communicate through other channels [31].

The microstructure of subchondral bone was shown by micro-CT in our study. Our findings are in accordance with studies reporting decreased Tb.Th in early and advanced OA [32]. However, Yuan et al. [33] and other scholars [34] have noted thickening of trabecular bone in early OA. Verborgt and coworkers stated that microinjuries to subchondral bone and calcified cartilage can initiate a repair mechanism, with activation of osteoclasts and osteoblasts [35]. In our study, measurements of the parameters of subchondral trabecular bone implied that osteoclast activity was greater than osteogenesis upon OFO. Subchondral bone is an effective shock absorber [36]. Once it becomes brittle, the lower efficiency of transferring loads from cartilage induced by resorption of subchondral bone, articular cartilage (which is a poor shock absorber due to its biologic characteristics) is vulnerable to damage [37]. Consequently, remodeling of subchondral bone fails to compensate for normal physiologic function under OFO, which can impair articular cartilage further.

The SDF-1-CXCR4 axis has been demonstrated to impair articular cartilage by inducing expression of pro-inflammatory cytokines and MMPs [38]. Liu and colleagues showed that SDF-1 can regulate osteogenic differentiation [39]. Yang and coworkers reported that increased expression of SDF-1 can impair the development and differentiation of osteoblasts [40]. We discovered that SDF-1 was expressed mainly in bone marrow, whereas CXCR4 was present in the hypertrophic layer. We found that the continuous force applied by OFO increased mRNA and protein expression of SDF-1 and CXCR4, caused cartilage degradation (with increased release of the tissue-destructive enzyme MMP13), and reduced expression of collagen II in the experimental groups compared with age-matched controls. The EXP + AMD3100 group showed strong resorption of subchondral bone but mild degradation of articular cartilage, compared with that in the EXP + NS group. Furthermore, IDS of OSX and SDF-1 revealed that SDF-1 expression was derived mainly from osteoblasts. These results suggested that increased expression of SDF-1 derived from osteoblasts could not only increase osteogenic differentiation but may also enable SDF-1 to bind CXCR4 to cause cartilage degradation. Using ADM3100 to inhibit SDF-1 signaling could reduce osteogenesis in subchondral bone and attenuate expression of MMP13.

## Conclusions

We found that OFO induced TMJ OA in rats. Obvious abnormal changes in subchondral bone may proceed to microscopic cartilage damage. Microinjuries induced by overloaded forces will cause enhanced activity of osteogenesis and osteoclasts, which could reduce the efficiency of transferring loads from cartilage due to brittle subchondral bone and damaged articular cartilage. SDF-1 derived from osteoblasts can increase osteogenic differentiation and induce MMP13 expression to cause cartilage degradation. However, the underlying mechanisms of TMJ OA induced by OFO must be investigated by focusing on intracellular signaling.

## Data Availability

All data generated and analyzed during this study are included in this published article.

## Abbreviations

OFO: Overloaded functional orthopedics
TMJ: Temporomandibular joint
TMJ OA: temporomandibular joint osteoarthritis
SDF-1: Stromal cell-derived factor-1
MMP13: Matrix metalloproteinase 13
CXCR4: CXC chemokine receptor 4
EXP: Experimental
NS: Normal saline
IDS: Immunofluorescence double staining
